# Genome-Wide Association Study for Susceptibility to and Recoverability From Mastitis in Danish Holstein Cows

**DOI:** 10.3389/fgene.2018.00141

**Published:** 2018-04-24

**Authors:** B. G. Welderufael, Peter Løvendahl, Dirk-Jan de Koning, Lucas L. G. Janss, W. F. Fikse

**Affiliations:** ^1^Department of Animal Breeding and Genetics, Swedish University of Agricultural Sciences, Uppsala, Sweden; ^2^Center for Quantitative Genetics and Genomics, Department of Molecular Biology and Genetics, Aarhus University, Aarhus, Denmark

**Keywords:** dairy cow, genome-wide association study, mastitis, recoverability, susceptibility

## Abstract

Because mastitis is very frequent and unavoidable, adding recovery information into the analysis for genetic evaluation of mastitis is of great interest from economical and animal welfare point of view. Here we have performed genome-wide association studies (GWAS) to identify associated single nucleotide polymorphisms (SNPs) and investigate the genetic background not only for susceptibility to – but also for recoverability from mastitis. Somatic cell count records from 993 Danish Holstein cows genotyped for a total of 39378 autosomal SNP markers were used for the association analysis. Single SNP regression analysis was performed using the statistical software package DMU. Substitution effect of each SNP was tested with a *t*-test and a genome-wide significance level of *P*-value < 10^-4^ was used to declare significant SNP-trait association. A number of significant SNP variants were identified for both traits. Many of the SNP variants associated either with susceptibility to – or recoverability from mastitis were located in or very near to genes that have been reported for their role in the immune system. Genes involved in lymphocyte developments (e.g., *MAST3* and *STAB2*) and genes involved in macrophage recruitment and regulation of inflammations (*PDGFD* and *PTX3*) were suggested as possible causal genes for susceptibility to – and recoverability from mastitis, respectively. However, this is the first GWAS study for recoverability from mastitis and our results need to be validated. The findings in the current study are, therefore, a starting point for further investigations in identifying causal genetic variants or chromosomal regions for both susceptibility to – and recoverability from mastitis.

## Introduction

Because of considerable improvements of production traits and unfavorable genetic correlations, mainly with milk production, mastitis continues to be one of the most frequent dairy cattle diseases with significant economic implications (Halasa et al., 2007; Hogeveen et al., 2011). Breeding can be instrumental in improving resistance to mastitis (Rupp and Boichard, 2003). However, genetic response to traditional selection is small because of the low heritability (*h*^2^ ≈ 0.03) (Heringstad et al., 2000; Carlén et al., 2004) of the trait. Moreover, mastitis is not easy to measure directly and often the phenotype data are binary (absent or present) at the observable scale. In genetic evaluations, this all-or-none trait definition may not fully utilize all information available in the data, for instance the duration and levels of infection (Carlén et al., 2005; Vazquez et al., 2009). Genome-wide association studies (GWAS) present options for direct selection of such traits through genetic markers. The results from GWAS can then be used in designing breeding schemes that increase the frequency of favorable alleles (Tiezzi et al., 2015).

Genome-wide association studies utilize information on genetic markers like single nucleotide polymorphisms (SNPs) to determine association with a trait of interest assuming that a marker is in linkage disequilibrium (LD) with, or close to, a causative mutation (Hirschhorn and Daly, 2005; Goddard and Hayes, 2009). In cattle, GWAS have been performed to evaluate marker or SNP association with clinical mastitis (CM) and with milk somatic cell count (SCC) as indicator trait, and many quantitative trait loci (QTLs) have been reported. QTLs for SCC have been reported on: *Bos taurus* autosomes (BTA) 6, 13, 14, and 20 in Nordic Holstein cattle (Sahana et al., 2013); BTA 6, 10, 15, and 20 in Irish Holstein-Friesian cattle (Meredith et al., 2012); 24 chromosomes (a total of 171 significant SNPs) in Valdostana Red Pied cattle breed (Strillacci et al., 2014); and on BTA 6, 13, 19 and X in German Holstein cows (Abdel-Shafy et al., 2014). A review (Sender et al., 2013) indicated that QTLs for mastitis resistance measured via either SCC or less frequently using CM records have been found on almost all chromosomes. The cattle QTL database^1^ also showed that QTLs for the keyword ‘mastitis,’ i.e., QTLs for mastitis related traits including CM, SCC or other indicator traits are more or less evenly spread over all chromosomes with the largest number of detected associations on BTA6 (see Supplementary Figure S1). BTA6 is known for harboring QTLs that affect both milk quality traits, e.g., the casein gene cluster (Nilsen et al., 2009; Pirola et al., 2013) and CM (Nilsen et al., 2009; Sodeland et al., 2011).

These studies have been useful for identifying genetic variants and genes associated with CM and indicator traits. However, they lack consistency in reporting SNPs or genes implicated in mastitis, and to our knowledge, none of these studies have reported any genetic variant or chromosomal regions associated with recoverability from mastitis. We, therefore, performed GWAS using single SNP regression analysis for two main objectives: (1) to identify new or confirm previously identified regions of the genome for their association with mastitis susceptibility, and (2) identify variants and regions of the genome associated with recoverability from mastitis in Danish Holstein cows.

## Materials and Methods

The raw phenotype data, analyzed in the current study, were edited and used by Welderufael et al. (2017) to estimate genetic parameters for susceptibility to – and recoverability from mastitis in Danish Holstein cows using bi-variate threshold models. The data contained a total of 89,232 weekly transition records for a total of 1,791 Danish Holstein cows in parities 1–3 distributed over one research herd (Danish Cattle Research Center, Tjele, Denmark) and six commercial dairy herds. Welderufael et al. (2017) derived the transition records from SCC data recorded at every milking using voluntary milking system (VMS, DeLaval International AB, Tumba, Sweden) and stored in an online database. Mastitis could be caused by different pathogens to which the cows may respond differently. However, such pathogen-specific data were not available in the current study. Welderufael et al. (2017) used the concept of transition model to define the traits of interest (susceptibility to – and recoverability from mastitis). The transition model helps to capture the variations an individual cow may exhibit in the entire disease course by capturing susceptibility as well as recovery. During a specified period of time, a cow is assumed to move between or within two states – healthy (H) and diseased (D). A cow may return to the same state more than once, meaning repeated disease cases are acknowledged by the model (Franzén et al., 2012). For a healthy cow, there is a risk of becoming infected and for a mastitic cow there is a possibility of recovering, which is conceptualized into probabilities of mastitis and recovery (Franzén et al., 2012). For each cow, several transitions from H to D, denoted as HD, and from D to H, denoted as DH, can occur within a lactation. Franzén et al. (2012) depicted such occurrences in a transition probability matrix, *T*_i_, for cow *i*, as follows:

$$T_{i}=\left[\begin{array}{cc} 1-\pi_{i}^{HD} & \pi_{i}^{HD}\\ \pi_{i}^{DH} & 1-\pi_{i}^{DH} \end{array}\right]$$

where $1-\pi_{i}^{HD}$ = Probability of remaining in the H state for cow *i.*

$\pi_{i}^{HD}$ = Probability of moving from H to D state for cow *i.*

$\pi_{i}^{DH}$ = Probability of moving from D to H state for cow *i.*

$1-\pi_{i}^{DH}$ = Probability of remaining in the D state for cow *i*.

In the above transition probability matrix, the first row consists of the probabilities of being in either of both states at time *t+*1for cow *i* that is healthy at time *t*, and the second row consists of the probabilities of being in either of both states at *t*+1 for cow *i* that is diseased at time *t*. In practice, the transition matrix is desired to have high values of $1-\pi_{i}^{HD}$ (probability of remaining in the H state for cow *i*) and $\pi_{i}^{DH}$ (probability of moving from D to H state, i.e., fast recovery if cow *i* had moved from H to D state), and consequently low values of $\pi_{i}^{HD}$ (probability of moving from H to D state) and $1-\pi_{i}^{DH}$ (probability of remaining in D state) (Franzén et al., 2012). Note that the probabilities sum to 1 within rows, and only two probabilities (two traits) are needed to model the transitions of animals between the H and D states.

Thus, for each cow and lactation the sequence of H’s and D’s, indicating whether or not a cow had mastitis on subsequent test weeks, was converted into a new sequence of weekly transitions indicators: 0 if a cow remains in the same state and 1 if the cow changes state. This resulted into two series of transition records: one for healthy to diseased (HD, to define susceptibility to mastitis) and the other for diseased to healthy (DH, to define recoverability from mastitis). A more detailed description of the data is given by Welderufael et al. (2017).

### Phenotypic Statistical Analyses

Phenotypic statistical analyses were performed to obtain adjusted phenotypes. The raw phenotypes (transition records) were adjusted for different fixed and random effects before using them in the association analysis. Welderufael et al. (2017) modeled the phenotype data as a linear combination of systematic effects and as a function of time. A lactation curve [*f*(DIM)] was modeled by a combination of Legendre polynomials and a Wilmink term (exp^-0.05×DIM^) (Wilmink, 1987), to reflect that susceptibility and recovery is not constant during the lactation. A time variable was used to indicate the duration of an episode. Changes of risk during each episode [*f*(time)] were modeled with Legendre polynomials. The model for the phenotypic analyses included parity (1–3) and herd (1–7) as fixed effects, regression coefficients of a second order polynomial on DIM plus a Wilmink term and regression coefficients of a third order polynomial on time as covariates, and random effects of herd-test-week, cow-parity interaction, and cow effects. The observed transitions were linked to an underlying continues scale called liability (Gianola and Foulley, 1983). This relationship between the observed binary response (y), and the unobservable liability (λ) is formally expressed as:

$$y=\left\{ \begin{array}{cc} 1, & if\,\;\lambda >\tau \\ 0, & if\,\;\lambda \leq \tau \end{array}\right.$$

where τ is a fixed threshold and usually set to an arbitrary value (here 0), such that *y* = 1 if λ > 0 and 0 otherwise. The mean on the liability scale models the average probability for *y* = 1. Accordingly, the observed transitions and liabilities were modeled with a threshold model as follows:

P(y=1)=P(λ>0)and

$$\lambda =Xb+Z_{1}h+Z_{2}p+Z_{3}c+e,$$

where λ was vector of the underlying liabilities linked to the transition scores (*y*); *b* was a vector of all fixed effects (herd and parity) and covariates (regression coefficients of a second order polynomial on DIM plus a Wilmink term and regression coefficients of a third order polynomial on time); *h*, *p*, and *c* were vectors of random effects of herd-test-week, cow-parity interaction, and cow effects, respectively; *X*, *Z*_1_, *Z*_2_, and *Z*_3_ were respective incidence matrices, *e* was a vector of the residual effects. The vectors of random effects (*h*, *p*, *c*, and *e*) were assumed to be normally distributed, i.e., $h∼N(0,\sigma_{htw}^{2}I),p∼N(0,\sigma_{cp}^{2}I),c∼N\left(0,\sigma_{c}^{2}I\right)$ and $e∼N\left(0,\sigma_{e}^{2}I\right)$, where $\sigma_{htw}^{2}$, $\sigma_{cp}^{2}$, $\sigma_{c}^{2}$, and $\sigma_{e}^{2}$ were, respectively, variances for herd-test-week, cow-parity interaction, cow, and residual effects, and *I* were the identity matrices. The residual variance ($\sigma_{e}^{2}$) is not identifiable, and it was fixed to 1. More detailed definitions and descriptions of the model and traits are provided by Welderufael et al. (2017).

The phenotypic analyses were performed using the statistical software package MCMCglmm (Hadfield, 2010) in R (R Core Team, 2016). Estimation of the random cow plus cow^∗^parity effects were based on 5,000 samples from a single chain of 250,000 iterations with a burn-in of 50,000 and samples stored at every 50^th^ round. In some association analyses, in particular the so called GRAMMAR analysis (Aulchenko et al., 2007), residuals from a polygenic model are taken as phenotypes. This procedure aims to pre-correct the data for polygenic effects. However, our association analyses include a random genetic (cow) effects in the model and the aim of the phenotypic analysis is to extract meaningful phenotypes for the GWAS. The posterior means and standard deviations of the random cow plus cow^∗^parity effects were obtained from the sum, mean and standard deviation of the estimates for each animal and trait. The calculated posterior means were used as adjusted phenotype data in the association analyses. The weights used in the association analyses were calculated as weight = 1/(PSD)^2^, where PSD is the posterior standard deviation of the cow plus cow^∗^parity effects. Association analyses were carried out on four datasets (each parity separately and all parities together) to investigate parity specific associations. The number of records, means and SD of the adjusted phenotype used in the association analyses are in **Table 1**.

**Table 1:** Number of records and mean (SD) used as phenotype in the association analyses for susceptibility to – and recoverability from mastitis.

	Susceptibility		Recoverability	
		
Parity	Number of records	Mean^1^ (*SD*)	Number of records	Mean^1^ (*SD*)
1	879	-0.01 (0.39)	471	0.00 (0.25)
2	719	-0.01 (0.39)	471	0.00 (0.24)
3	451	-0.02 (0.37)	325	-0.01 (0.22)
All	2049	-0.01 (0.39)	1267	0.00 (0.24)


*^*1*^The adjusted mean phenotypes were calculated from the sum of cow plus cow^∗^parity effects for each trait.*

Further bivariate and trivariate analyses were performed to study correlations between traits as well as between parities but within traits. The bivariate model was similar to equation (2) and except the residual, all between traits random effects were assumed correlated. The trivariate model used to calculate correlations between parities was also similar to the model in equation (2) but did not include parity and cow-parity interaction effects. In this trivariate model only the cow effects across parities were correlated. Herd-test-week effects were included and assumed uncorrelated.

### Genotype Data

The raw data contained 1957 cows genotyped with the Illumina BovineSNP50_v2 (Illumina Inc., San Diego, CA, United States) for a total of 46931 autosomal SNP markers. However, we use phenotypes records from our previous study (Welderufael et al., 2017) and the animals with phonotypes records in our previous study were not all genotyped. In our previous study (Welderufael et al., 2017), we had phenotypes records for a total of 1791 cows. After matching the phenotype to the genotype data, only 997 cows with phenotypes remained in the genotype data. Quality control (QC) for markers was done using PLINK (Purcell et al., 2007). SNPs with more than 10% missing genotypes and SNPs with minor allele frequency (<1%) were excluded. One cow was also removed from next analyses for having less than 90% genotype call rate. All the 39378 SNP markers used for the final association analyses were distributed over the cattle genome as shown in Supplementary Figure S2.

### Detection of Population Stratification

Multidimensional scaling (MDS) of SNP genotypes was performed for detection of population stratification for the 996 animals that passed genotyping quality control checks. The aim was to assess potential genetic clustering of animals by herd. The cows came from seven herds: one research herd and six commercial herds. In order to visualize possible population stratifications, MDS plots of an identity-by-state (IBS) matrix was generated using PLINK (Purcell et al., 2007).

**FIGURE 1:**
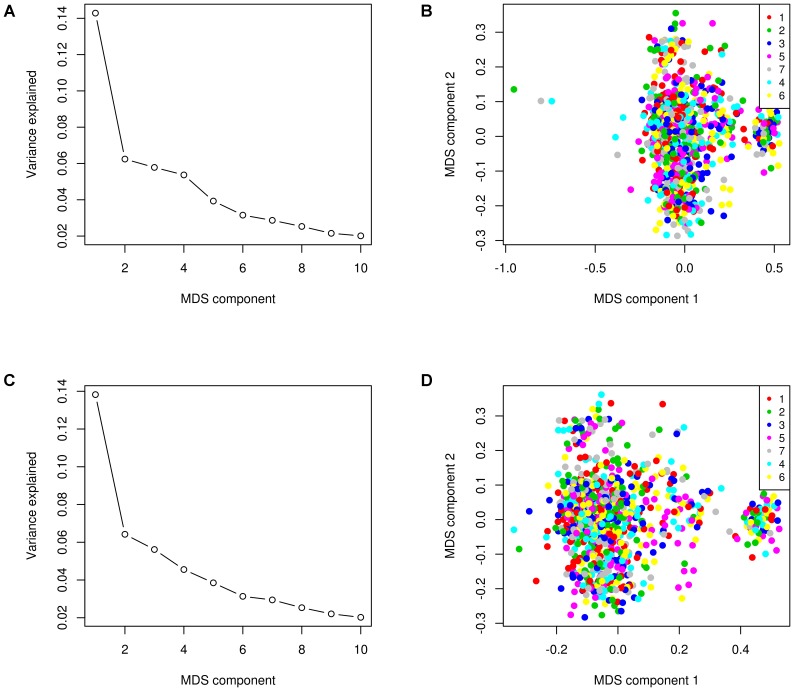
Multidimensional scaling (MDS) plot of identity-by-state (IBS) matrix. Genomic variance explained by the first 10 MDS components before **(A)** and after **(C)** removing genetic outlier animals. The MDS plot before **(B)** and after **(D)** removing the three genetic outlier animals shows the population structure within herds (distinguished by color) identified by the first two dimensions.

As shown in **Figures 1A** and **1C**, the first five MDS components explained 14.3, 6.2, 5.8, 5.4, and 3.9%, respectively, of the total genomic variance, and therefore, we reasoned that fitting these first five MDS components as clustering factor into our model could improve the GWAS results because it corrects for confounding effect of the population structure (Price et al., 2006). The MDS plots of the IBS matrix further revealed that three animals were genetic outliers and appeared to form their own distinct cluster (**Figure 1B**). After removing the three genetic outlier animals, the remaining animals formed no distinct cluster (**Figure 1D**).

After QC and removal of four cows (one for having less than 90% genotype data and three other cows for being genetic outliers), 993 cows and 39378 SNP markers remained and were used for final association analyses.

### Association Analysis and Significance Test

Single SNP association analysis was performed with a linear mixed model in DMU (Madsen and Jensen, 2013). Each SNP was fitted as a covariate. To account for shared genetic effects of related individuals, pedigree-based polygenic effect was included by fitting individual animal as a random effect in the model. To account for the confounding effect of population structure observed in the MDS plots, the top five MDS components were included as covariates in the association analysis. The single SNP regression analysis was performed using the following statistical model:

y=1μ+Xb+Sα+Za+e

where *y* was a vector of posterior mean of summed cow and cow^∗^parity effects obtained from equation (2); 1 was a vector of ones; μ was the general mean; *X* was a matrix containing the top five MDS components fitted as covariates, and *b* was a vector of associated effects; *S* was vector of SNP genotypes coded as 0, 1, or 2 for genotype copies of one of the alleles at each locus; α was the allele substitution effect; *a* was a vector of random additive polygenic effects, which was assumed to follow normal distribution, $a∼N\left(0,A_{\sigma_{a}^{2}}\right)$ where *A* is the pedigree-based additive genetic relationship matrix; *Z* was an incidence matrix relating elements of the vector of additive polygenic values *a* to individual phenotypes; *e* was a vector of random residuals and the residuals were assumed to follow a normal distribution, $e∼N\left(0,W\sigma_{e}^{2}\right)$, where *W* was a diagonal matrix with diagonal element $w_{ii}=1/{\left(PSD\right)}_{1}^{2}$; $\sigma_{a}^{2}$ and $\sigma_{e}^{2}$ were the additive polygenic and residual variances, respectively. The weight *w*_i_ is used to account for the different residual variances due to parities. The pedigree for the animals (993) in the genotype data was extended to a maximum depth of 10.9 generations and an average of 4.4 known generations. The extended pedigree contained a total of 12779 animals and were offspring of 2659 sires and 9536 dams.

The association analysis was conducted using the statistical software package DMU (Madsen and Jensen, 2013). The null hypothesis *H*_0_: b = 0 was tested with a *t*-test. A genome-wide Bonferroni corrected significance threshold can be obtained by dividing the nominal significance threshold (0.05) by the number of independent tests (total number of SNPs, 39378) in the analyses. The *P*-value after Bonferroni multiple testing corrections was 1.26974E-06, equivalent to -log_10_ (*P*-value) = 5.90. However, in the current study no *P*-value exceeded the Bonferroni threshold and hence a liberal threshold of *P*-value < 10^-4^ was used to declare significant SNP-trait associations.

### Candidate Gene Identification

Candidate genes were identified by checking whether significantly associated SNPs were located within a gene, or within 1 Mb up- or downstream from a gene using the Ensembl^2^ gene annotation system (Aken et al., 2016). We also made use of the human gene database- GeneCards^3^ for functional annotations of those identified genes. Throughout the text, genomic positions of genetic variants and genes were based on the *Bos taurus* genome UMD3.1 assembly (Zimin et al., 2009).

## Results

### Descriptive Statistics of the Phenotypic Analysis

Herd-test-week variation was lower for the HD trait than for the DH trait (**Table 2**), which translates to less variation in susceptibility to- than in recoverability from mastitis within year across time. On the other hand, animal related variation was higher for susceptibility to mastitis (HD) than for recoverability from mastitis (DH) (**Table 2**). The between traits posterior mean of cow effect correction [*r*_c_ = -0.90 (0.07)] was much higher than the cow-parity interaction correction [*r*_cp_ = -0.29 (0.11)].

**Table 2:** Posterior mean (SD) of variances and correlations between susceptibility to – (HD) and recoverability (DH) from mastitis, and between parities but within traits.

Item	Cow effect			Cow^∗^parity effect			Herd-test-week effect		
			
	Mean (*SD*)	Lower 95%CI	Upper 95%CI	Mean (*SD*)	Lower 95%CI	Upper 95%CI	Mean (*SD*)	Lower 95%CI	Upper 95%CI
**Variance**									
$\sigma_{HD}^{2}$	0.13 (0.02)	0.10	0.17	0.18 (0.02)	0.15	0.22	0.02 (0.00)	0.01	0.03
$\sigma_{DH}^{2}$	0.10 (0.02)	0.06	0.15	0.12 (0.03)	0.07	0.18	0.06 (0.01)	0.04	0.09
**Between traits correlation**									
r_(HD, DH)_	-0.90 (0.07)	-0.99	-0.77	-0.29 (0.11)	-0.50	-0.07	0.08 (0.17)	-0.24	0.40
**Between parities (within traits) correlation**									
HD									
*r*_P12_	0.44 (0.07)	0.31	0.56	–^1^	–	–	–^2^	–	–
*r*_P13_	0.08 (0.12)	-0.14	0.30	–	–	–	–	–	–
*r*_P23_	0.44 (0.08)	0.29	0.60	–	–	–	–	–	–
DH									
*r*_P12_	0.52 (0.17)	0.18	0.88	–	–	–	–	–	–
*r*_P13_	0.43 (0.28)	-0.07	0.93	–	–	–	–	–	–
*r*_P23_	0.10 (0.20)	-0.30	0.47	–	–	–	–	–	–


**r*_*P12*_ = correlation between parity 1 and 2.*

**r*_*P13*_ = correlation between parity 1 and 3.*

**r*_*P23*_ = correlation between parity 2 and 3.*

*^*1*^The cow^∗^parity effect is not relevant in the parity specific analyses.*

*^*2*^The herd-test-week effect is not correlated in the parity specific analyses.*

The variance components presented in **Table 2** also showed large cow-parity interaction effects, which indicates these traits to be very parity specific. To show this even clearer we added new parity specific analyses for the phenotype input variable, which confirmed low to medium correlations (0.08–0.52) across parities. As expected, for the HD trait, the smallest between parities cow effect correlation was observed between the non-consecutive parities 1 and 3 [*r*_p13_ = 0.08 (0.12)], whereas for the DH trait, the smallest between parities cow effect correlation was observed between the consecutive parities 2 and 3 [*r*_p23_ = 0.10 (0.20)] (**Table 2**).

### SNP Variants Associated With Susceptibility to – and Recoverability From Mastitis

The nominal significance level of *P*-value < 10^-4^ was considered to determine genome-wide significant SNP-trait association. For the full data (all parities analyzed together), significant SNP-trait associations were detected on BTA3 and 7 for mastitis susceptibility and on BTA7 and 15 for recoverability from mastitis (**Table 3**). Parity specific significant SNP-trait association were also detected on the same chromosomes but no overlapping association signals were observed. Significant association signals in parity 1 and in the other parities (parity 2 or 3) detected on the same chromosome did not overlap, for either of the traits. For susceptibility to mastitis, the highest number of significant associations were observed on BTA7 and BTA13. For recoverability from mastitis, the highest number of significant associations were observed on BTA12 and BTA13. In total, 29 significant SNPs (14 for susceptibility to mastitis and 15 for recoverability) were detected.

**Table 3:** List of SNPs in the *Bos taurus* autosomes (BTA) showing significant [-log10(*P*-value) > 4] associations with susceptibility to – (HD) and recoverability (DH) from mastitis.

Trait^∗^	BTA	SNP rsID	α	SEα	-log_10_*P*	Nearest gene(s)	Candidate gene	Distance (Kb)^∗∗∗^
HD	3	rs109583509	-0.07	0.02	4.31	GRIK3	GRIK3	90112
	7	rs43732911	-0.24	0.06	4.09	MARCH3	MARCH3	Overlap
DH	7	rs29012637	-0.05	0.01	4.44	EPS15L1	EPS15L1	79088
	15	rs110414316	-0.09	0.02	5.13	STARD10, ATG16L2, FCHSD2	ATG16L2	Overlap
	15	rs109344144	-0.08	0.02	5.45	P2RY2, P2RY6, ARHGEF17	P2RY6	4544
	15	rs42991413	-0.09	0.02	4.31			
P1HD	1	rs109029759	-0.10	0.03	4.20	PLOD2	PLOD2	269678
	4	rs110921945	0.14	0.03	4.07	ENSBTAG00000047979	ENSBTAG00000047979	Overlap
	8	rs109438746	-0.10	0.03	4.10	SCARA3, TMEM215, ENSBTAG00000005574	SCARA3, ENSBTAG00000005574	6358
	12	rs41631671	-0.17	0.04	4.30	CYSLTR2, FNDC3A		
P1DH	15	rs42550814	0.09	0.02	4.87	DDI1, PDGFD	PDGFD	Overlap
	20	rs41638346	0.15	0.04	4.08	IPO11	IPO11	226699
P2HD	5	rs109785134	0.15	0.03	5.21	STAB2, NT5DC3	STAB2	Overlap
	6	rs42639714	0.10	0.03	4.30	PCDH7	PCDH7	434135
	9	rs42574126	0.11	0.03	4.02			
P2DH	1	rs109126926	0.08	0.02	4.11	VEPH1, PTX3	VEPH1, PTX3	Overlap
	6	rs43317449	-0.09	0.02	4.49	LRIT3, RRH, GAR1, CFI	LRIT3	5431
	6	rs43453628	0.08	0.02	4.85	SEC24B, COL25A1	COL25A1	291654
	13	rs109674956	-0.10	0.02	4.93	PREX1	PREX1	20117
	26	rs41648638	-0.33	0.08	4.05	WDR11	WDR11	112161
P3HD	7	rs41255569	-0.14	0.04	4.25	IFI30, MAST3, ENSBTAG00000002350	IFI30, MAST3	1375
	12	rs41618899	0.12	0.03	4.04	FREM2, TRPC4	FREM2	278734
	13	rs43150474	0.16	0.04	4.67	SLX4IP, JAG1	SLX4IP	Overlap
	13	rs109036286	-0.13	0.03	4.31			
	28	rs29012361	-0.13	0.03	4.08			
P3DH	3	rs41612148	-0.26	0.06	5.39	ENSBTAG00000047248	ENSBTAG00000047248	182592
	3	rs41626429	-0.26	0.06	4.95	ENSBTAG00000047248	ENSBTAG00000047248	339288
	5	rs43145932	-0.14	0.04	4.20	ATXN7L3B	ATXN7L3B	678832
	7	rs41565600	-0.10	0.02	4.17	CSNK1G3	CSNK1G3	271378


**^∗^Trait: HD and DH represent susceptibility to – and recoverability from mastitis all parities analyzed together; P1HD, P2HD, and P3HD = susceptibility to mastitis for parity 1, 2, and 3, respectively; P1DH, P2DH, and P3DH = recoverability from mastitis for parity 1, 2, and 3, respectively.**

**^∗∗^The SNP positions in base pairs (bp) were based on the UMD3.1 genome assembly (ftp://ftp.cbcb.umd.edu/pub/data/assembly/Bos_taurus/Bos_taurus_UMD_3.1/).**

**^∗∗∗^Distance = physical distance (Kb) from the SNP to the start/end of the nearest candidate gene*.*

**FIGURE 2:**
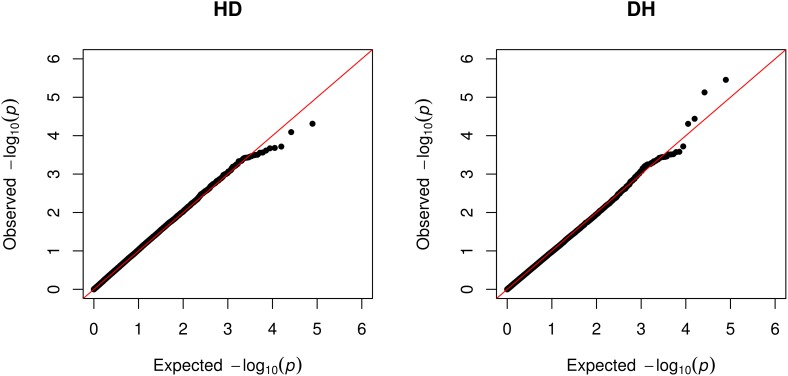
QQ-plot of the expected null distribution of the *P*-values vs. the observed null distribution of the *P*-values for susceptibility to – (HD) and recoverability (DH) from mastitis for all parities analyzed together.

**FIGURE 3:**
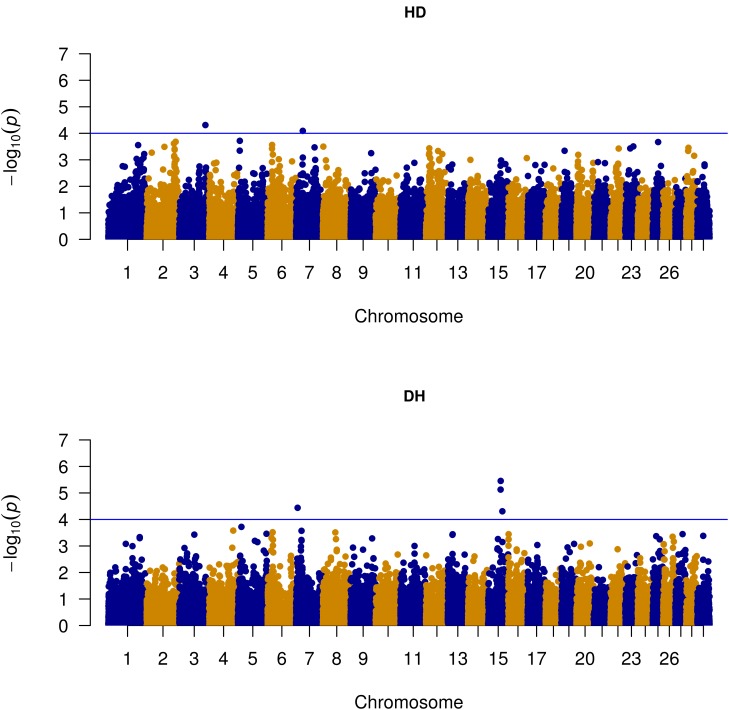
Manhattan plot of genome-wide associations with susceptibility to – (HD) and recoverability from mastitis (DH) for all parities. The blue line represents suggestive significance level [–log_10_(*P*-value) = 4].

As shown in the quantile–quantile (QQ) plot (**Figure 2**) and Manhattan plot (**Figure 3**), only 6 SNPs (2 for susceptibility and 4 for recoverability) reached the defined genome-wide significance threshold when all parities were analyzed together. The QQ plots and Manhattan plots for the parity specific associations are provided in the Supplementary Material (Supplementary Figures S3–S8). The clear deviation from the expected *P*-value only in the tail area of the QQ plots (**Figure 2** and QQ plots in the Supplementary Material) further demonstrated that the GWAS results were not substantially influenced by the observed population stratification. Influence of population stratification was adequately controlled by including the MDS components in the model and this was quantified by a calculated genomic inflation factor ≤ 1.05 (on average λ = 1.02, results not shown).

## Discussion

Because mastitis is very frequent and unavoidable, adding recovery information to the analysis is of great interest. In this study we have performed GWAS using single SNP regression analysis and identified SNP variants in and near by possible causative genes not only for susceptibility to – but also for recoverability from mastitis. To our knowledge, this is the first study to report associated SNP variants and identify possible causative genes for recoverability from mastitis in Danish Holstein cows.

### Variances and Correlations From the Phenotypic Analyses

The lower posterior mean of herd-test-week variance for HD than for DH could indicate that management to prevent mastitis (and other factors influencing susceptibility) is fairly stable across time, whereas management (and other factors) related to recoverability is more variable over time. This could suggest that there are possibilities to enhance recoverability by improvements in herd management (Welderufael et al., 2017). The posterior mean of cow related variances indicates less variation in recoverability from mastitis than the variation in susceptibility to mastitis among cows. Between traits, the high posterior mean of cow effect correlation (*r*_c_ = -0.90) indicates that highly susceptible cows are in general less likely to recover fast. Relatively, the trait correlation between cow-parity interaction effects was much smaller than the cow effect correlation, indicating even highly susceptible cows in parity one could recover relatively faster in the other next parity.

The cow effect correlations across parities but within traits were positive and ranged from low to moderate. For the HD trait, the weakest posterior mean of cow effect correlation across parities was observed between parities 1 and 3, indicating that susceptibility in parity 1 is distinct from susceptibility in parity 3. For the DH trait, the weakest posterior mean of correlation across parities was observed between parities 2 and 3, suggesting recoverability in parities 2 and 3 should not be considered as the same trait. In general, we would expect parities to be similar, but this data shows larger variation in cow-parity interaction than the cow effect variation as we presented it in **Table 2**. The cow-parity interaction variance is larger than the cow variance, i.e., the larger part of cow variance is parity specific. Results from the trivariate analysis where parities are considered as different traits, also point to relatively low agreement between parities (**Table 2**). So, the data does not support the general expectation that parities may be similar, and this can explain the absence of common association signals across parities in the association results. A possible confounding effects of multiple mastitis pathogens may have contributed to the lack of consistent association signals across parities and traits. Sørensen et al. (2009) reported that genetic correlations among pathogen-specific mastitis traits are far from 1 (ranged from 0.45 to 0.77), suggesting that mastitis caused by different pathogen should be considered as different traits.

### SNP Variants Associated With Susceptibility to – and Recoverability From Mastitis

Mastitis is a complex trait. Numerous functional candidate genes could be involved in both directions of the disease, susceptibility to – and recoverability from mastitis. In the current study, most of the significant association signals in parity 1 and in the other parities (parity 2 or 3) detected on the same chromosome do not overlap, suggesting that genetic regulation of mastitis could also be parity dependent. Effect of genes has been shown to vary depending on parity or age of cows (Wojdak-Maksymiec et al., 2013). Wojdak-Maksymiec et al. (2013) reported that allele T of the gene *TNF-*α was associated with a lower number of mastitis cases in lower parities and a higher number of mastitis cases in higher parities. Similarly, the causal variants or genes could be different for susceptibility to – and recoverability from mastitis as association signals present on the same chromosome do not overlap. Our results support the findings that QTLs even for highly correlated traits (e.g., SCC and CM) present on the same chromosome do not overlap (Sender et al., 2013). Most of the significant variants discovered in the current study for both susceptibility to – and recoverability from mastitis lies within or nearby genes that have been annotated for their role in immune systems. But we observe no overlap of signals neither across parities nor traits. Here, therefore, we discuss a few potential candidates for causal genes separately for parities and traits.

Most of the suspected candidate genes for susceptibility to mastitis are found in chromosomes that are frequently reported (e.g., on the cattle QTL data base) for their association with mastitis traits defined from SCCs. The SNP rs43732911 on BTA7, an intron variant and in significant association with susceptibility to mastitis in the full dataset, is located within a potential candidate protein coding gene *MARCH3* (E3 ubiquitin-protein ligase MARCH3). The gene *MARCH3* encodes an E3 ubiquitin-protein ligase enzyme that may be involved in regulation of the endosomal transport pathway and it has been implicated in diverse biological functions, such as immune regulation, protein quality control, and membrane trafficking (Fukuda et al., 2006). The SNP rs109785134 on BTA5, an intron variant in significant association with susceptibility to mastitis in parity 2, is located within a protein coding gene *STAB2* (stabilin-2 precursor). Studies in pigs (Howard et al., 2015) and in mice (Schledzewski et al., 2011) showed that the gene *STAB2* is involved in clearing of metabolic waste from blood circulation. The protein encoded by this gene functions in angiogenesis, lymphocyte homing, cell adhesion, or receptor scavenging (GeneCards; D’Souza et al., 2013). The receptor has been shown to bind and endocytose ligands such as hyaluronan, low density lipoprotein, Gram-positive and Gram-negative bacteria, and advanced glycosylation end products (GeneCards). The SNP rs109785134 on BTA13, a missense variant and in significant association with susceptibility to mastitis in parity 3, is located within the protein coding gene *SLX4IP* (*SLX4* interacting protein). *SLX4*-deficient mice have been shown to develop epithelial cancers and to have a contracted hematopoietic stem cell pool (Hodskinson et al., 2014).

Most of the candidate genes for recoverability from mastitis identified by the intron variants have been reported for their association with biological processes of adaptation and immune systems. The SNP rs110414316 on BTA15, an intron variant and in significant association with recoverability from mastitis in the full dataset is located in a protein coding gene *ATG16L2* (autophagy related 16 like 2). The gene *ATG16L2* has been reported to play a role in autophagy (Yin et al., 2014). The SNP rs42550814 on BTA15:4668361, an intron variant and in significant association with recoverability from mastitis in parity 1, is located within a protein coding gene *PDGFD* (platelet derived growth factor D). The protein encoded by the gene *PDGFD* plays an important role in wound healing and macrophage recruitment (Uutela et al., 2004). In a transgenic mice experiment (Uutela et al., 2004), the gene has been shown to induce macrophage recruitment, to elevate interstitial fluid pressure in dermis and resulted to wound healing. Even though, the type of wound is not from bacterial infection like as is in mastitis, the gene plays a significant role in wound recovery and we think we have found a possible candidate for causal gene in recoverability. The SNP rs109126926 on BTA1, an intron variant and in significant association with recoverability from mastitis in parity 2, is located within a protein coding gene *PTX3* (pentraxin 3). The gene *PTX3* may involve in the innate immune response against intra-amniotic infection and inflammation (Cruciani et al., 2010). The expression of this protein is induced by inflammatory cytokines in response to inflammatory stimuli in several mesenchymal and epithelial cell types, particularly endothelial cells and mononuclear phagocytes (GeneCards). Cytokines, an important group of inflammatory mediators, play an important role in the host innate immune response to infection (Fu et al., 2013). The protein encoded by the gene plays a role in angiogenesis and tissue remodeling which is very important part of recovery, and the protein serves as a biomarker for several inflammatory conditions (GeneCards). Lutzow et al. (2008) found *PTX3* to be upregulated after *Staphylococcus aureus* infection, which is one of the main pathogens for mastitis. This gene has been reported in several studies for its role in innate immune response and regulation of inflammation (Lutzow et al., 2008; Brenaut et al., 2014). A review (Garlanda et al., 2016) on gene targeted mice and genetic associations in humans suggest that *PTX3* plays an important role in resistance against different pathogens including *Escherichia coli*. Therefore, we suggest that this gene is a possible candidate for the causal gene in recoverability from mastitis.

We have also identified some potential candidate genes from the nearby gene cluster, in addition to the genes identified by within gene SNP variants. The SNP rs41255569 on BTA7, an upstream gene variant and in significant association with susceptibility to mastitis in parity 3, is mapped very near to a functionally known candidate genes *MAST3* (microtubule associated serine/threonine kinase 3) and *IFI30* (lysosomal thiol reductase). Both genes have been reported for their role in antigen processing. In a gene expression assays (Labbe et al., 2008), the gene *MAST3* showed abundant expression in antigen-presenting cells and in lymphocytes. It has been also shown that knockdown of *MAST3* decreased the presence of Toll-like receptor-4-dependent NF-kappaB that initiate the innate immune response during pathogen invasion (Labbe et al., 2008). The enzyme (a lysosomal thiol reductase) encoded by the gene *IFI30* has also an important role in MHC class II-restricted antigen processing (GeneCards). The enzyme also facilitates MHC class I-restricted recognition of exogenous antigens containing disulfide bonds by CD8+ T-cells or cross-presentation (GeneCards).

**FIGURE 4:**
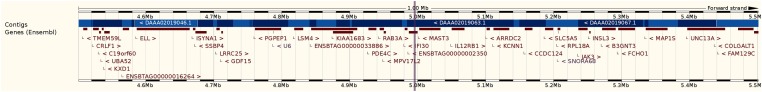
1 Mb gene clusters on BTA7 surrounding significantly associated rs41255569 variant (located at about 5.00 Mb) with susceptibility to mastitis in parity 3.

In **Figure 4** we present a 1Mb gene cluster surrounding the significantly associated variant in the region. This illustrates that complex traits could be regulated by several clusters of genes surrounding association signals and confirms the fact that mastitis is a complex trait and is determined by many genes with small effect limiting the ability of GWAS to identify causal genes (Hayes et al., 2010).

Similarly, the SNP rs29012637 on BTA7, an intergenic variant, was significantly associated with recoverability from mastitis, and is mapped very near to the gene *EPS15L1* (epidermal growth factor receptor pathway substrate 15 like 1). The gene *EPS15L1* has been reported as an essential gene for T lymphocyte development in Zebrafish (Seiler et al., 2015). As the gene is highly conserved between zebrafish, mouse and human protein, it is likely to have similar functions in other mammals (Seiler et al., 2015), including cattle. Therefore, the gene *EPS15L1* could be considered as a potential candidate gene for recoverability from mastitis because it plays an important role in promoting production of lymphocytes, which are main components in the immune system.

Biscarini et al. (2016) used an alternative approach to detect regions of the genome associated with susceptibility to infectious and metabolic diseases in dairy cows. Their approach was based on runs of homozygosity (ROH), where stretches of homozygous DNA are compared in cases and controls and those associated with susceptibility to disease are supposed to be more frequent in cases than in controls. The SNP rs41618899 in the current study and in significant association with susceptibility to mastitis in parity 3 is located within a previously reported ROH [stretching from 25878820 to 30099199 bps on BTA12 (Biscarini et al., 2016)] associated with infectious diseases including mastitis. Similarly, the SNP rs41565600 in significant association with recoverability from mastitis in parity 3 is located within a previously reported ROH [stretching from 2991449 to 4655753 bps on BTA7 (Biscarini et al., 2016)] associated with different infectious diseases including mastitis.

From the annotations related to immune responses, it is plausible to suspect *MARCH3* (involved in regulation of the endosomal transport pathway), *MAST3* (highly expressed in antigen-presenting cells and in lymphocytes) and *STAB2* (involved in lymphocyte homing, cell adhesion, and receptor scavenging) as candidate for the causal genes for susceptibility to mastitis. For recoverability from mastitis we suggest *EPS15L1* (essential for lymphocyte development), *PDGFD* (involves in macrophage recruitment and wound healing), and *PTX3* (involved in regulating inflammation) as candidates for the causal genes. Regardless of the gene annotations related to immune responses, we were not fully convinced to suggest these genes as strong candidates as we were not able to confirm previously identified SNP variants or regions of the genome. However, this is the first GWAS study for recoverability from mastitis and our results need to be validated. Moreover, most of the functional annotations were derived from gene expression studies in mice and humans and gene functions may be different in cattle. There have also been discrepancies between gene-expression platforms and analyses methods reported to be obstacles in mapping loci that might underlie functional trait variation (de Koning and Haley, 2005). The findings in the current study could be considered as a starting point for further investigations in identifying causal genetic variants or chromosomal regions for both susceptibility to – and recoverability from mastitis and especially for the latter as this is the first study to report SNP variants and chromosomal regions.

## Conclusion

This study is the first in reporting SNP variants for their association with recoverability from mastitis in Danish Holstein cows. We have identified 29 significant SNP variants (14 for susceptibility to – and 15 for recoverability from mastitis) determined at a significance level of *P*-value < 10^-4^. Among these detected SNP variants, most of them are in genes known for their role in immunity and wound healing. On the bovine genome, locations of association signals were different for different parities, suggesting that effect of genes are parity dependent. Similarly, association signals for mastitis susceptibility were mapped in different locations from associations for recoverability from mastitis, suggesting that susceptibility to – and recoverability from mastitis are regulated by different genes.

## Author Contributions

BW performed the genome-wide association analysis and wrote the draft manuscript. PL and LJ contributed to the research hypothesis and discussion of the results. D-JK contributed to the research hypothesis, analysis, discussion of the results, and writing the manuscript. WF contributed to the research hypothesis, analysis of phenotype data, and discussion of the results. All authors approved the final version of the manuscript.

## Conflict of Interest Statement

The authors declare that the research was conducted in the absence of any commercial or financial relationships that could be construed as a potential conflict of interest.

## Footnotes

1 http://www.animalgenome.org/cgi-bin/QTLdb/BT/index, accessed September 2017

2 https://www.ensembl.org

3 http://www.genecards.org
