# Supplementary material for: Genome-Wide Association Study for Susceptibility to and Recoverability From Mastitis in Danish Holstein Cows
Source: Front Genet. 2018 Apr 24;9:141. doi: 10.3389/fgene.2018.00141 (PMC5932407; doi:10.3389/fgene.2018.00141)
Supplement: Supplementary file 1 [file Data_Sheet_1.DOCX]

Supplementary Material

Genome-wide Association Study for Susceptibility to- and Recoverability from Mastitis in Danish Holstein Cows

B.G. Welderufael^1,2*^, P. Løvendahl^2^, D.J. de Koning^1^ , L.L.G. Janss^2^ and W.F. Fikse^1^

^1^Department of Animal Breeding and Genetics, Swedish University of Agricultural Sciences,

Uppsala, Sweden

^2^Center for Quantitative Genetics and Genomics, Department of Molecular Biology and Genetics, Aarhus University, Tjele, Denmark

***Correspondence:** Corresponding Author: [berihu.welderufael@slu.se](mailto:berihu.welderufael@slu.se)

# Supplementary Figures


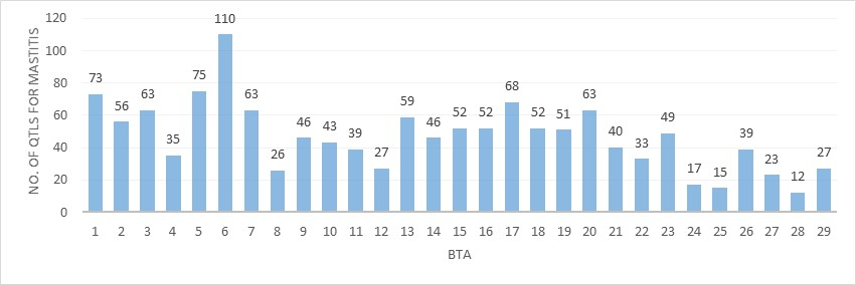


**Supplementary Figure 1**. Number of quantitative trait loci (QTLs) for mastitis over the cattle genome (<http://www.animalgenome.org/cgi-bin/QTLdb/BT/index>, accessed September 05, 2017).


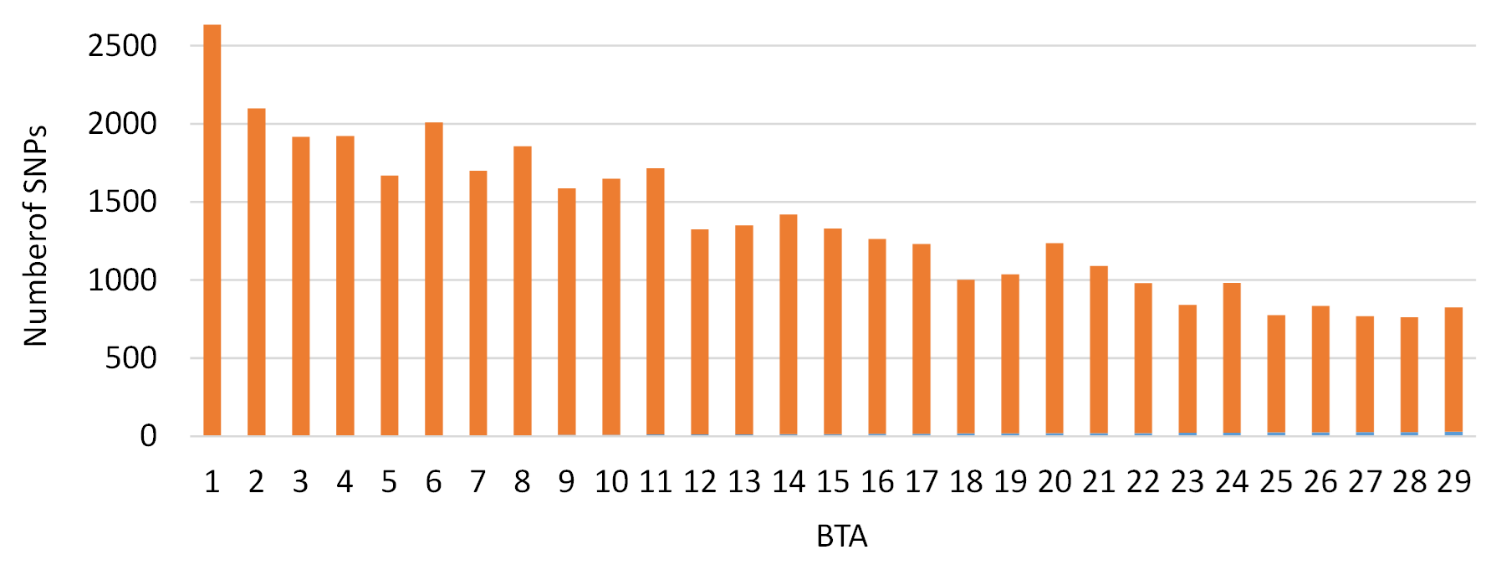


**Supplementary Figure 2**. Distribution of the 39378 SNPs (after QC) over the *Bos taurus* autosomes (BTA).


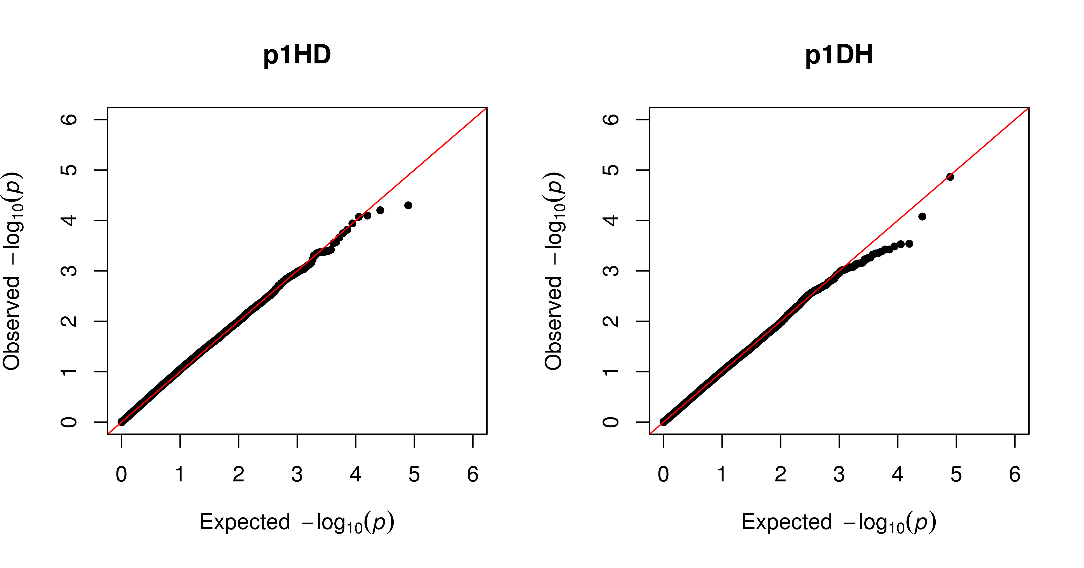


**Supplementary Figure 3.** QQ-plot of the expected null distribution of the p-values versus the observed null distribution of the p-values for susceptibility to- (right) and recoverability from (left) mastitis for parity 1.

**
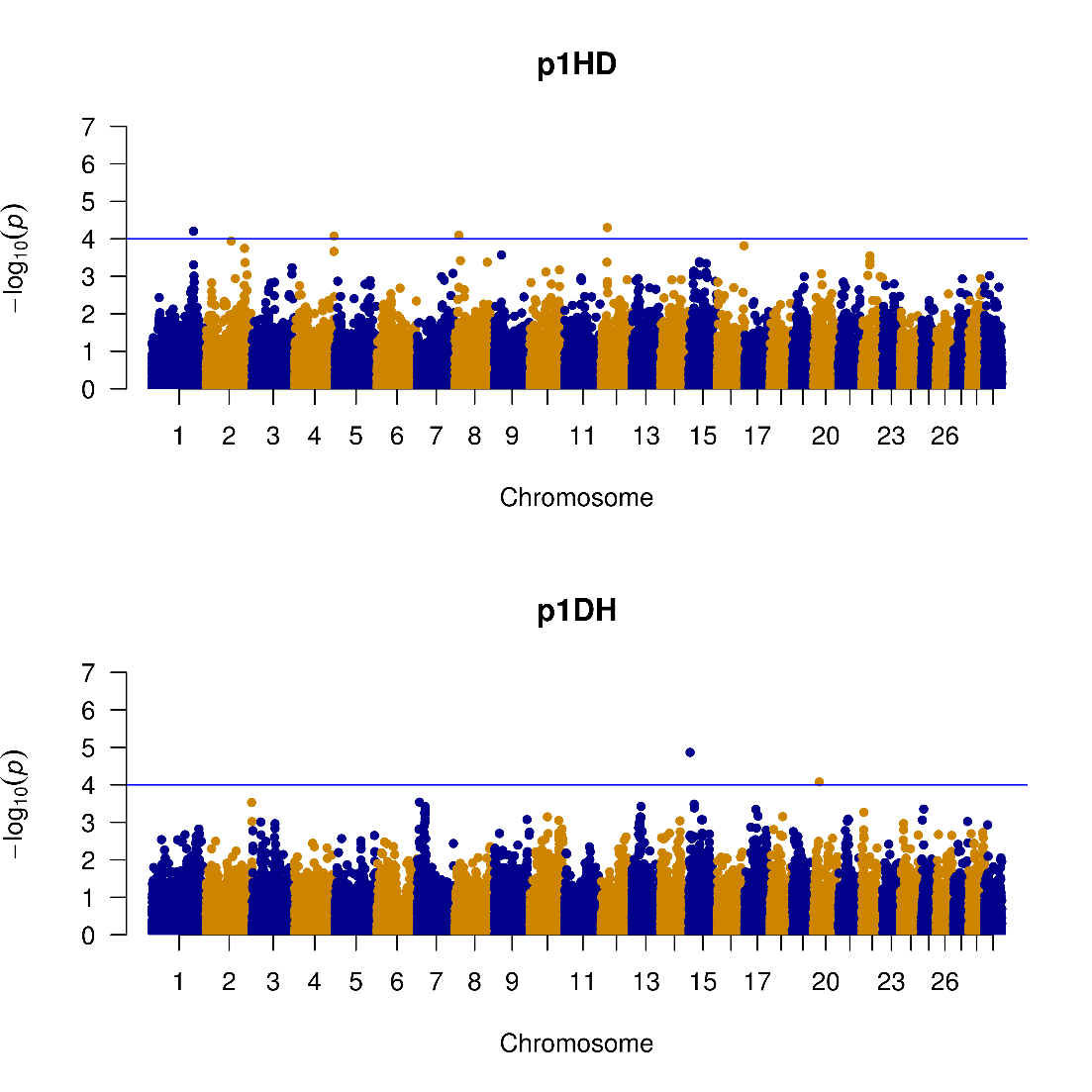
**

**Supplementary Figure 4.** Manhattan plot of genome-wide associations with susceptibility to- (top) and recoverability from mastitis (bottom) for parity 1. The blue line represents suggestive significance level [–log_10_(p-value) = 4].


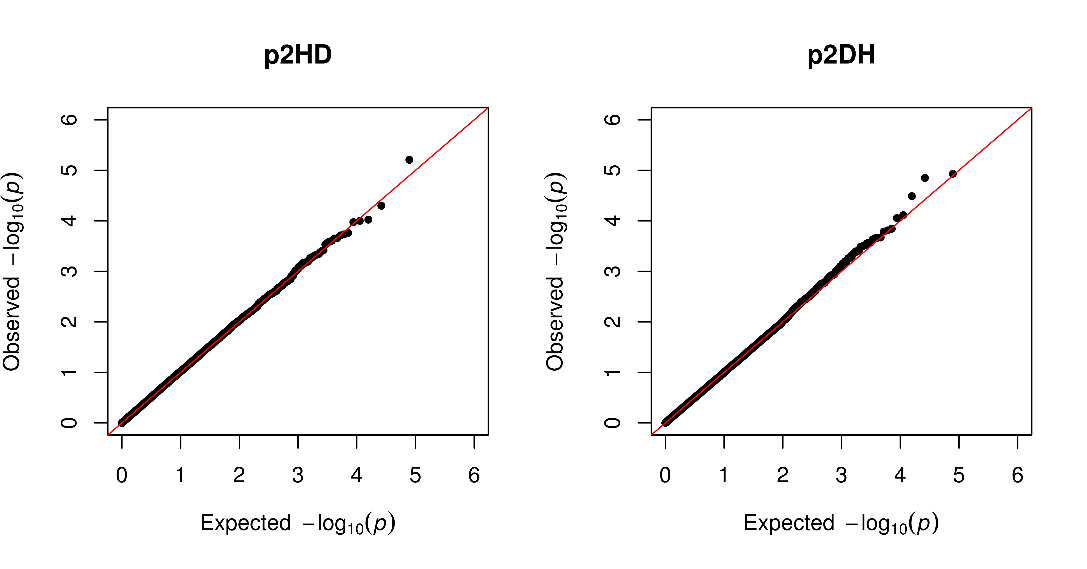


**Supplementary Figure 5.** QQ-plot of the expected null distribution of the p-values versus the observed null distribution of the p-values for susceptibility to- (right) and recoverability from (left) mastitis for parity 2.


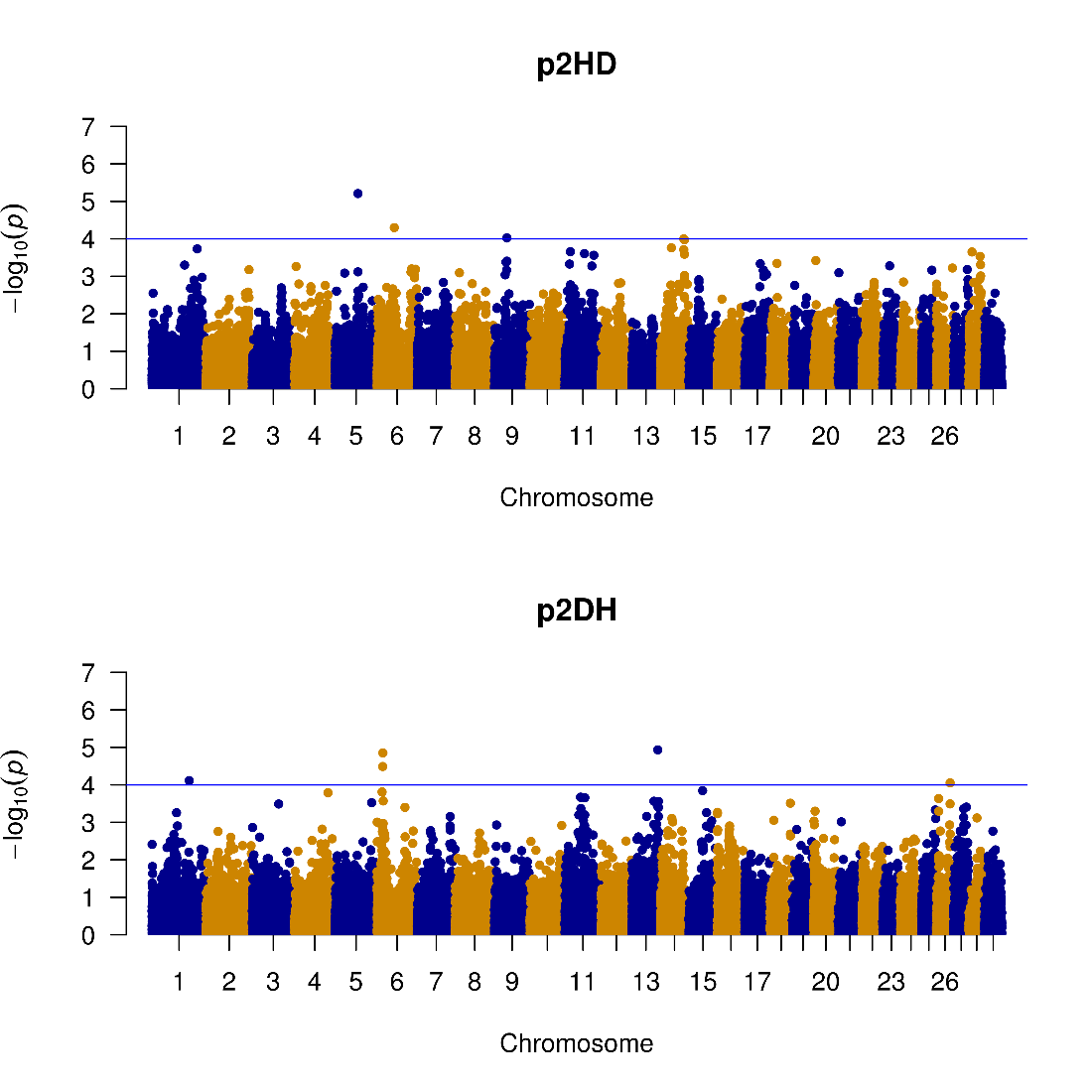


**Supplementary Figure 6.** Manhattan plot of genome-wide associations with susceptibility to- (top) and recovery from mastitis (bottom) for parity 2. The blue line represents suggestive significance level [–log_10_(p-value) = 4].


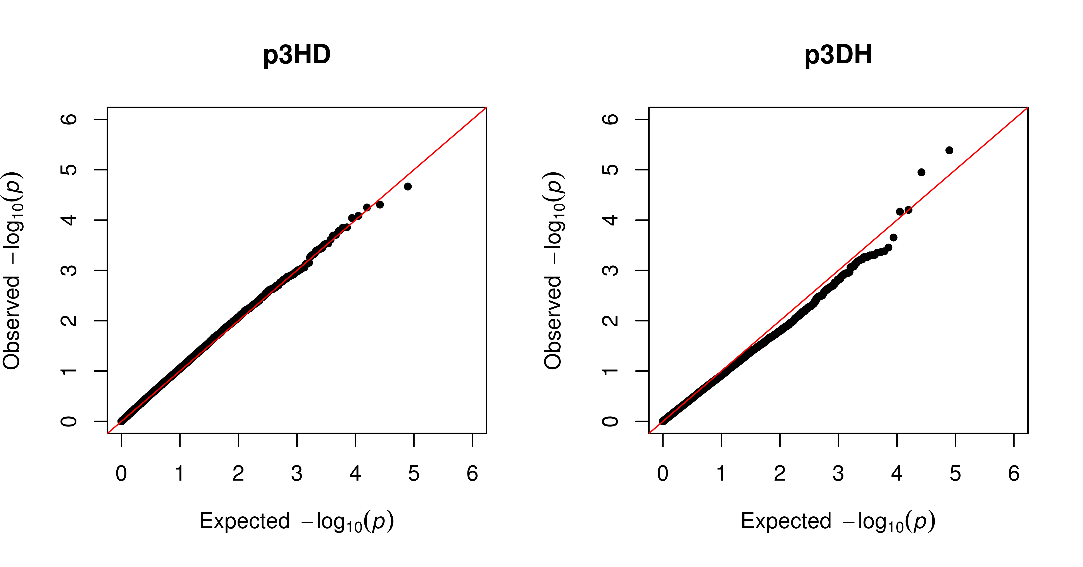


**Supplementary Figure 7.** QQ-plot of the expected null distribution of the p-values versus the observed null distribution of the p-values for susceptibility to- (right) and recoverability from (left) mastitis for parity 3.


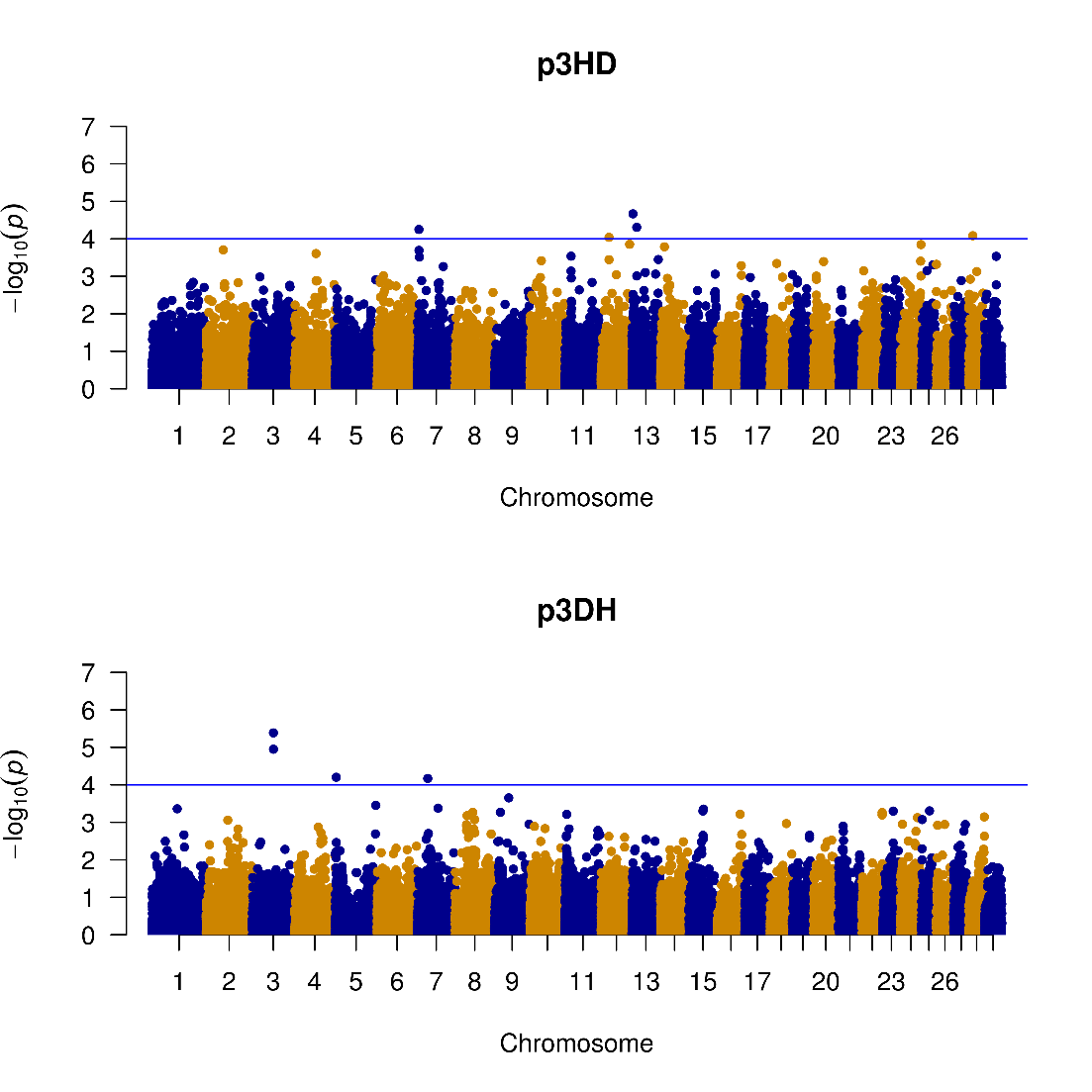


**Supplementary Figure 8.** Manhattan plot of genome-wide associations with susceptibility to- (top) and recovery from mastitis (bottom) for parity 3. The blue line represents suggestive significance level [–log_10_(p-value) = 4].
